# Carpometacarpal Joint Arthroplasty in a Patient With Concomitant Von Willebrand Disease: A Case Report

**DOI:** 10.7759/cureus.80501

**Published:** 2025-03-12

**Authors:** Oles Petrovych, Jakub Florek, Filip Georgiew, Adam Bębenek

**Affiliations:** 1 Department of Orthopaedics and Traumatology, Rydygier Hospital, Brzesko, POL; 2 Faculty of Medicine and Health Science, University of Applied Sciences, Tarnów, POL; 3 Department of Neurosurgery, St. Lucas Hospital, Tarnów, POL

**Keywords:** arthroplasty, carpometacarpal, cmc, endoprosthesis, osteoarthritis, thumb, von willebrand disease

## Abstract

Thumb carpometacarpal (CMC) joint osteoarthritis is characterized by severe pain, reduced grip strength, and restricted thumb mobility. This condition is among the most prevalent forms of arthrosis, significantly impacting patients' daily activities and overall quality of life. Managing patients with coagulation disorders presents a particular challenge, as they are at increased risk for postoperative bleeding, infectious complications, and the potential need for revision surgery. Von Willebrand disease (VWD) type I, a disorder caused by a deficiency of Von Willebrand factor (VWF), can lead to prolonged blood clotting times. This case report demonstrates the successful management of bilateral CMC joint osteoarthritis in a patient with VWD type I using arthroplasty, emphasizing the importance of perioperative hematological management to prevent complications.

## Introduction

The first carpometacarpal (CMC 1) joint is critical for thumb function, and its degeneration significantly impairs hand mobility and quality of life, particularly in patients with coagulation disorders like Von Willebrand disease (VWD). This joint has a unique saddle-shaped structure, where the articular surfaces alternate between concave and convex orientations in both anteroposterior and lateral projections. Its loose and inherently unstable structure allows for a wide range of movements, including flexion, extension, adduction, abduction, opposition, and rotation [1,2].

Osteoarthritis of the CMC joint of the thumb is among the most common degenerative joint conditions, particularly affecting the wrist and hand [3,4]. The disease predominantly affects women, with studies estimating that approximately 25% of postmenopausal women develop rhizarthrosis of the CMC joint. The prevalence of CMC joint osteoarthritis increases with age, particularly in postmenopausal women, making it a common cause of hand dysfunction [4,5].

There are no universally accepted protocols for selecting the optimal surgical treatment for thumb CMC rhizarthrosis. The choice of procedure is often guided by the surgeon’s experience and preference. Several surgical techniques are available, including CMC 1 joint arthrodesis, trapezium resection, and CMC 1 joint arthroplasty. All these methods are effective in reducing pain and improving joint mobility [6,7]. Arthroplasty, while relatively expensive due to implant costs, offers several advantages: preservation of knuckle column length, postoperative joint stability, early rehabilitation initiation, and faster functional recovery. As a result, arthroplasty is the most commonly performed surgical treatment for CMC 1 rhizarthrosis in Europe [7].

Von Willebrand disease is the most common congenital bleeding disorder, affecting approximately 1% to 2% of the population [8-13]. It accounts for about 20% of all congenital coagulation disorders in Poland, though the actual number may be higher due to undiagnosed cases [8,9]. First described in 1924 by Finnish physician Erik von Willebrand, the disease involves qualitative or quantitative defects in Von Willebrand factor (VWF), leading to impaired platelet adhesion and Factor VIII transport at sites of vascular injury [8,11]. Type I VWD, the most common form (70% to 80% of cases), is characterized by a quantitative deficiency of VWF. Clinical manifestations include frequent nosebleeds, heavy menstruation, post-traumatic bleeding, gum, and gastrointestinal bleeding, and an increased risk of postoperative complications such as excessive blood loss, infections, and joint hemorrhages, which can accelerate joint degeneration and arthrosis [10,13]. Surgical management of patients with VWD presents challenges, particularly in settings without access to a hematologist. These patients require thorough preoperative and postoperative preparation, including VWF and Factor VIII supplementation or desmopressin administration. Proper perioperative management significantly reduces the risks of bleeding, infections, and the need for revision surgery [10,13].

## Case presentation

**Figure 1:**
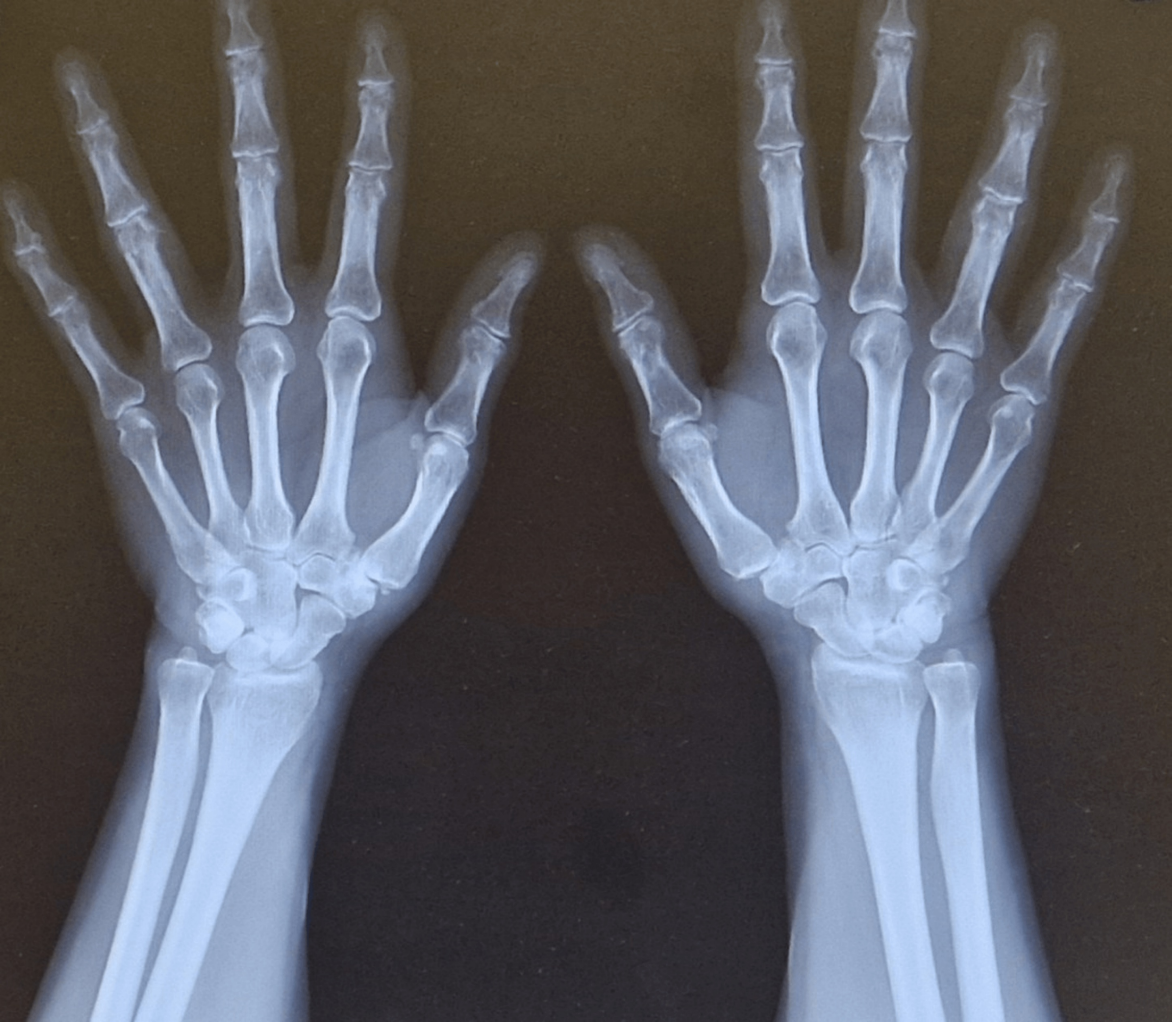
Preoperative X-ray in anteroposterior (AP) projection

**Figure 2:**
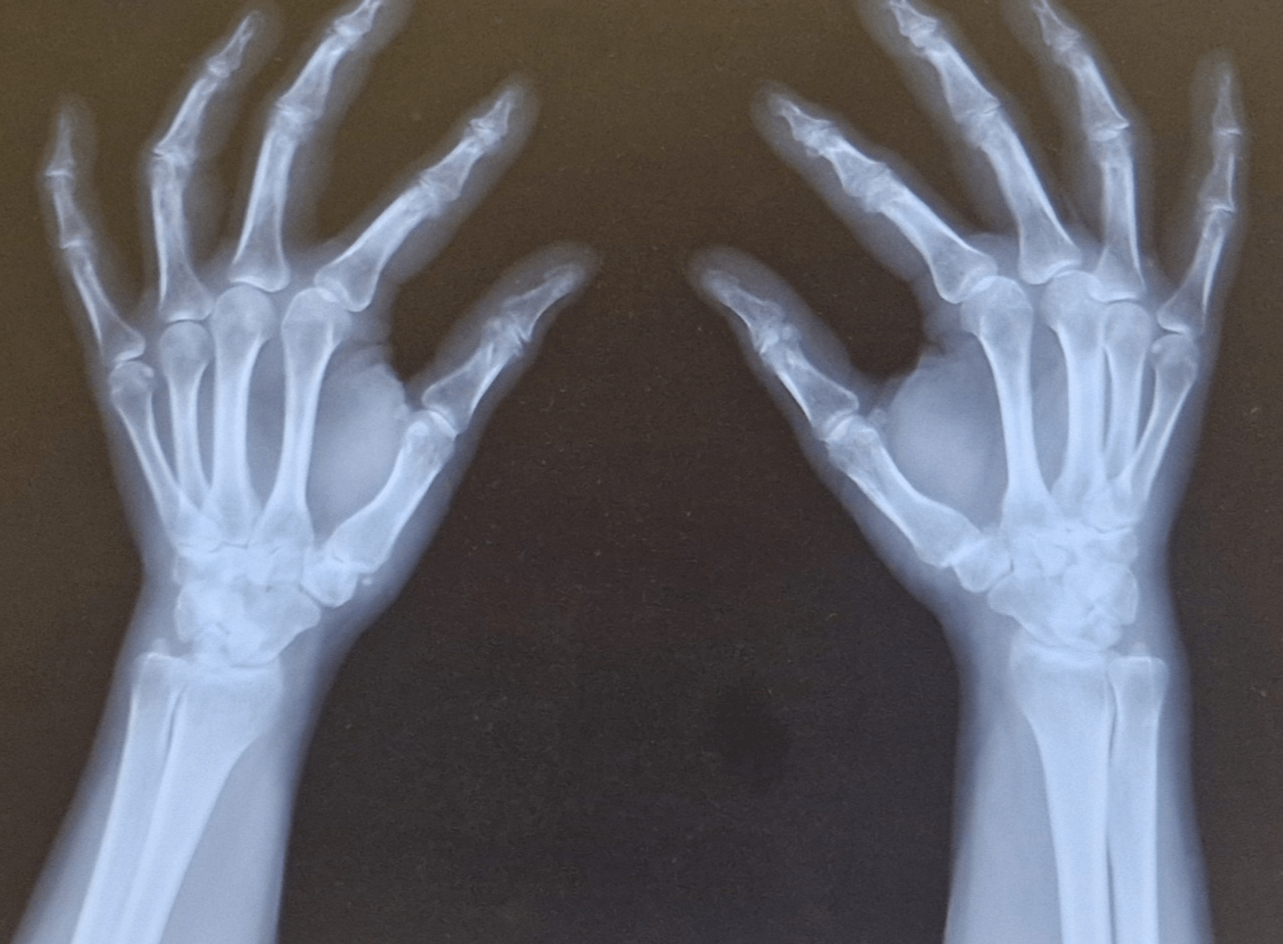
Preoperative X-ray in the Kapandji projection

We present the case of a 50-year-old female patient with VWD type I, who presented with severe pain in the anatomical snuffbox region and restricted thumb mobility bilaterally. These symptoms significantly affected her daily and professional activities. Radiographic evaluation (Figures 1-2), including anteroposterior (AP) and Kapandji projections, confirmed bilateral degenerative changes classified as stage II-III according to the Eaton-Littler/Glickel classification [14].

The clinical examination revealed the following findings in the patient's hands: Pain at rest in the right and left hands rated four on the Numeric Rating Scale (NRS); pressure pain in the right hand rated four and seven in the left hand on the NRS scale. Painless opposition range limitation (Kapandji scale, one to 10) in the right thumb was three, and in the left thumb was two (where one is the most limited and 10 is the normal range of motion (ROM)). The grinding test was positive as it showed pain with rotational pressure on the first metacarpal bone against the trapezium in both hands. The pressure-shear test was positive for pain with lateral movement of the first metacarpal base under pressure on the trapezium in both hands. The thumb stability test was positive, indicating subluxation of the CMC joint. Grip strength was weak in the right hand (36 pounds) and left hand (24 pounds). These findings confirm significant functional impairment and pain, consistent with the diagnosis of CMC joint osteoarthritis (rhizarthrosis) in both thumbs. The patient was qualified for arthroplasty using the Touch bimobile prosthesis (Keri Medical, Plan-les-Ouates, Switzerland). Due to VWD type I, a hematological consultation was conducted, and a perioperative treatment plan was established.

Hematological management

Fanhdi (Grifols, Barcelona, Spain), containing VWF and factor VIII, was administered: 3,000 units IV before surgery and 2,000 units IV twice daily for seven days postoperatively. The patient was also on Estrofem 2mg/day as hormone replacement therapy after hysterectomy. Daily laboratory tests were conducted throughout hospitalization to monitor coagulation parameters and overall condition. Table 1 presents the changes in key perioperative parameters during hospitalization, reflecting the patient’s response to treatment and recovery process.

**Table 1:** Changes in key laboratory parameters during subsequent days of hospitalization

Parameter	Reference range	Operational day							
		Before	0	1	2	3	4	5	6
White blood cell (WBC) K/mkl	4.0 – 10.0	4.62	5.58	4.68	4.2	4.58	3.86	4.63	4.5
Red blood cell (RBC) M/mkl	4.0 – 5.40	4.72	4.12	4.24	4.09	4.23	4.32	4.56	4.5
Hemoglobin (HGB) g/dl	11.0 – 16.0	13.6	12	11.8	11.4	12.3	12.5	13.1	13
Platelets (PLT) K/mkl	150.0 - 450.0	191	154	153	159	177	185	211	219
Prothrombin time (PT) seconds	12.0 - 18.0	10.8	10.5	10.2	10.3	9.7	10.3	10.4	11.3
International normalized ratio (INR)	0.79 – 1.25	1.01	0.98	0.95	0.96	0.9	0.96	0.97	1.06
Activated partial thromboplastin time (aPTT) seconds	22.70 – 32.50	29.5	27	25.3	26.7	28.1	29.1	30.4	31.2
Fibrinogen (FIBR) mg/dl	200.0 – 400.0	204	203	295	332	378	313	325	231

**Figure 3:**
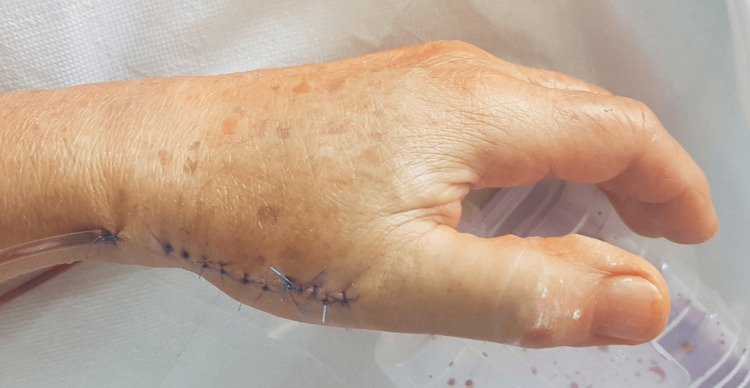
Postoperative wound

Due to greater dysfunction, the left hand was operated on first. Surgical treatment was performed using Esmarch ischemia to minimize intraoperative bleeding. A straight longitudinal incision was made over the CMC joint of the left hand, followed by careful dissection to expose the nerve bundle, which was left dorsally. The CMC joint capsule was cut longitudinally, and joint repair was performed on the first metacarpal bone and the greater trapezium bone. The insertion of the abductor pollicis longus (APL) tendon was cut off to facilitate access, and osteophytes were removed from the base of the first metacarpal bone. Using original instrumentation, a base was prepared for the prosthesis pin in the first metacarpal bone and a socket in the greater trapezium bone. After selecting the appropriate implant sizes, the prosthesis was implanted, and stability was confirmed, ensuring no luxation of the endoprosthesis elements. The implant position was verified using C-arm imaging. The procedure concluded with APL tendon reattachment, thorough hemostasis, and layered suturing. The thumb was then immobilized in a figure-of-eight plaster splint. Figure 3 shows the postoperative wound appearance.

**Figure 4:**
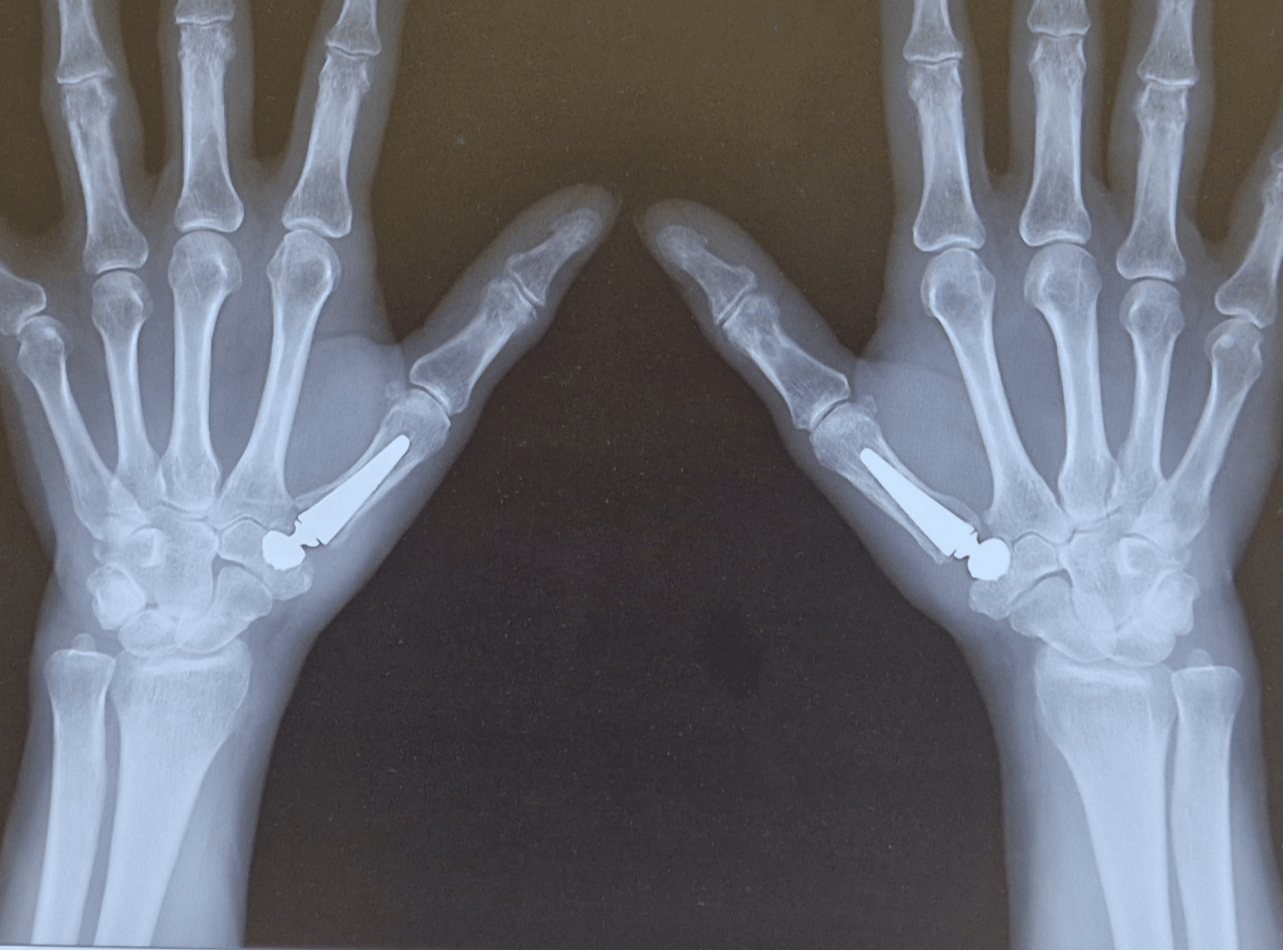
Postoperative anteroposterior (AP) X-ray

**Figure 5:**
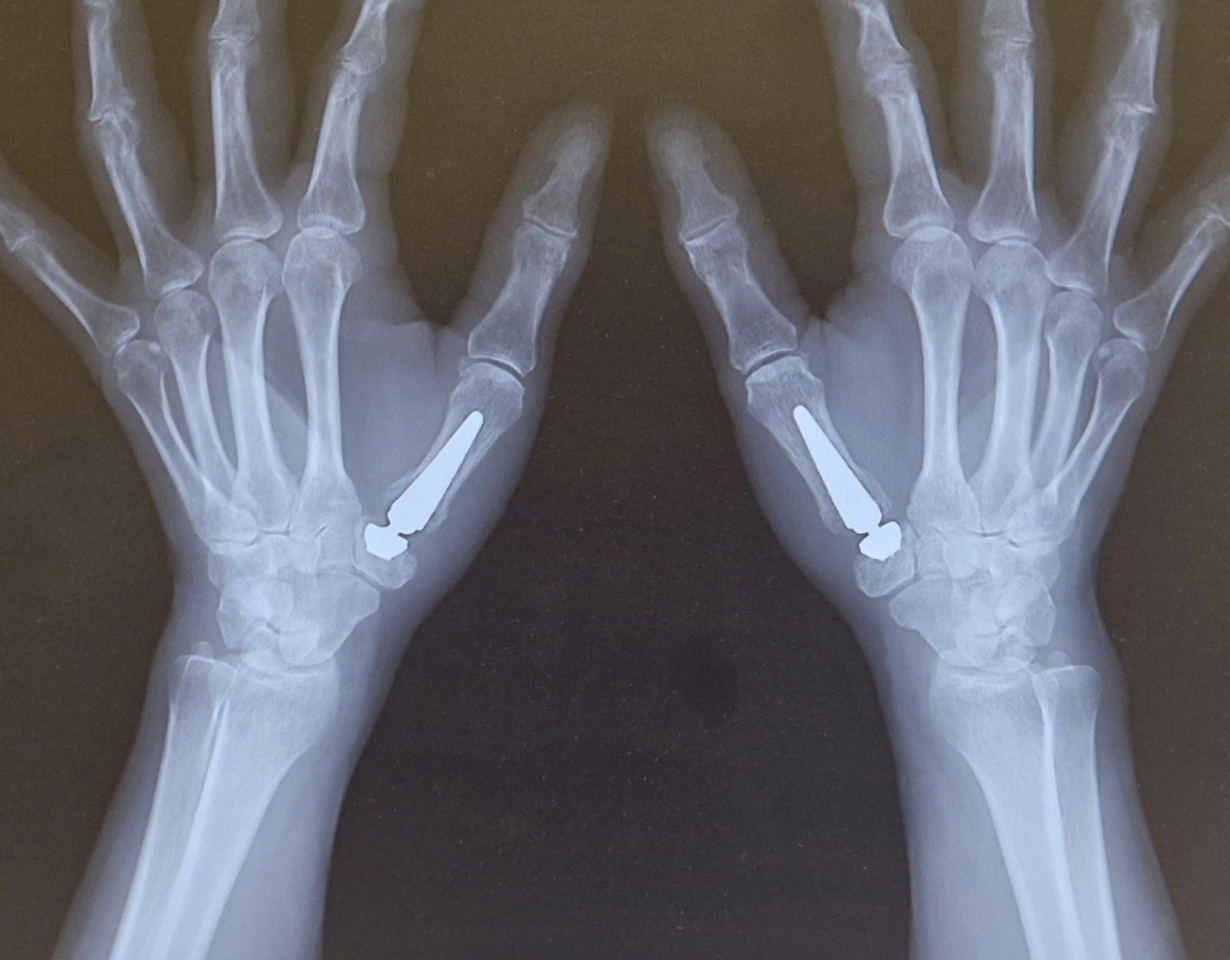
Postoperative X-ray in the Kapandji projection

Two weeks after the procedure, the stitches and immobilization were removed, and rehabilitation commenced. In the sixth week post operation, during the follow-up visit, an X-ray was taken, confirming the correct position of the implants. Three months after the procedure, the patient was able to return to professional activity. One year after the first procedure, another arthroplasty of the CMC 1 joint in the right thumb was performed. The surgical and hematological treatment followed the same protocol as for the left upper limb. Follow-up evaluations, six months after the right-hand procedure and 18 months after the left-hand procedure, revealed significant clinical improvements and no complications such as postoperative bleeding, infection, or luxation of the endoprosthesis elements. Clinical assessment showed the following: a reduction in resting pain for both hands, down to one on the NRS scale; a decrease in pressure pain around the right joint to two and around the left joint to 0 on the NRS scale; an increase in the painless range of opposition movement, with the right thumb achieving a value of eight and the left thumb reaching nine on the Kapandji scale; negative results for the grind test, pressure-shear test, and thumb stability test for both hands; and an improvement in global grip strength to 38 pounds for the right hand and 35 pounds for the left hand. Figures 4-5 present the radiological results obtained by the patient after surgical treatment, six months after the procedure (right hand), and 18 months (left hand).

## Discussion

Osteoarthritis of the CMC 1 joint constitutes a significant clinical challenge, particularly for women of working age, due to the associated severe pain and resultant impairment of physical function, which compromises daily activities. The absence of a standardized surgical protocol for CMC joint osteoarthritis complicates treatment decisions, particularly in patients with coagulation disorders like VWD, where bleeding risks must be carefully managed. However, the absence of a comprehensive classification system complicates the decision-making process regarding the most appropriate surgical approach for individual cases. Resection of the greater trapezius bone offers effective pain relief, though it results in a reduction in thumb length. Complete resection may lead to increased intraoperative trauma and postoperative bleeding, a concern of particular significance in patients with VWD. Arthrodesis of the CMC 1 joint also provides substantial pain relief but leads to the complete loss of joint mobility, which substantially impairs hand function. Thus, the choice of surgical technique is guided by the surgeon's expertise, patient-specific factors, and the need to minimize complications, particularly in high-risk populations such as those with coagulation disorders [6, 7]. 

Von Willebrand disease represents a considerable challenge in the surgical management of CMC 1 arthritis due to its association with blood coagulation disorders, which increase the risk of postoperative complications. As the prevalence of VWD and other coagulation disorders continues to rise, the importance of preoperative planning, hematological control, and the implementation of preventive strategies becomes paramount [8, 9]. In cases where CMC 1 arthrosis coexists with VWD, the collaborative involvement of a hematologist, along with the judicious selection of surgical techniques, can lead to favorable clinical outcomes while minimizing the risk of complications. While surgical intervention is essential for managing advanced CMC joint osteoarthritis, uncommon complications such as traumatic heterotopic ossification can arise, particularly in patients with predisposing factors like coagulation disorders or prolonged immobilization. The perioperative hematological management regimen employed in this case, which included treatment with Fanhdi and vigilant monitoring of blood counts, coagulation parameters, and fibrinogen levels, facilitated optimal hemostasis, supported effective postoperative healing, and contributed to an excellent clinical outcome [10-12].

## Conclusions

First carpometacarpal joint arthroplasty with the Touch bimobile prosthesis, combined with meticulous perioperative hematological management, resulted in significant clinical improvement in a patient with VWD type I, highlighting the importance of multidisciplinary care in high-risk surgical cases. The perioperative hematological management protocol employed facilitated proper healing of the postoperative wound and successfully prevented complications, demonstrating the importance of integrated care in managing complex cases.
